# Association of Late Marriage and Low Childbirth with Cervical Cancer Screening among Korean Women: Results from a Nationwide Survey

**DOI:** 10.3390/cancers14020327

**Published:** 2022-01-10

**Authors:** Hye Young Shin, Bomi Park, Mina Suh, Kui Son Choi, Jae Kwan Jun

**Affiliations:** 1 National Cancer Control Institute, National Cancer Center, Goyang 10408, Korea; hs103740@ncc.re.kr (H.Y.S.); omnibus@ncc.re.kr (M.S.); kschoi@ncc.re.kr (K.S.C.); 2 Department of Preventive Medicine, College of Medicine, Chung-Ang University, Seoul 06974, Korea; bpark@cau.ac.kr; 3 Graduate School of Cancer Science and Policy, National Cancer Center, Goyang 10408, Korea

**Keywords:** pap smear, mass screening, uterine cervical neoplasms, young adult

## Abstract

**Simple Summary:**

Marriage and childbirth may affect adherence to cervical cancer screening. We have examined whether marriage and childbirth were associated with the adherence to cervical cancer screening among young adult women in Korea. Among 3925 women aged 20–39 years, 39.1% undertook cervical cancer screening within two years of eligibility. Compared with unmarried women, married women were more likely to adhere cervical cancer screening (adjusted odds ratio = 2.80, 95% CI: 2.99–3.44). And, as the number of births in married women increased, the adherence to cervical cancer screening increased.

**Abstract:**

This study aimed to identify the association of marriage and childbirth with the adherence to cervical cancer screening among young adult women. Data across four years (2017–2020) of the cross-sectional Korean National Cancer Screening Survey were used. For measuring the adherence to cervical cancer screening, we used the cervical cancer screening rate with recommendation, which was defined as the percentage of women in the population eligible for screening who have had a cervical cancer screening within the past two years. Multiple logistic regression analysis was conducted to identify the association between marriage and adherence to cervical cancer screening. Overall, 3925 women aged 20–39 years were analyzed. Of these, 39.1% were screened for cervical cancer (26.6% unmarried and 57.1% married women). The married women had significantly higher adherence to cervical cancer screening than unmarried women (adjusted odds ratio = 2.80, 95% CI: 2.99–3.44). Compared with unmarried women, adherence to cervical cancer screening was significantly more likely to increase (*p* for trend, <0.001) in married women with an increased number of births. Our study confirmed that marriage and childbirth influence adherence to cervical cancer screening, suggesting that unmarried women may be vulnerable to cervical cancer.

## 1. Introduction

Recently, the incidence of cervical cancer has increased among young adult women in several countries [1]. In Korea, the incidence of cervical cancer among women aged 15–34 years was ranked third highest [2]. Among young adult women in Korea, changes in the culture around sexual behavior have been taking place rapidly over the past decade [3,4]. Sexual activity is now beginning earlier and becoming more frequent, increasing the chance of exposure to cervical cancer risk factors [4]. In response to these changes, the National Cancer Screening Program (NCSP) for cervical cancer in Korea, which provides a free conventional Papanicolaou smear (Pap smear) once every two years to women aged ≥30 years since 2002, has extended testing to women who are aged ≥20 years since 2016 [5].

Marriage and childbirth could provide a pivotal moment for exposure to and adherence to cervical cancer screening among young adult women [6]. However, in Korea, the marriage rate has dramatically decreased in recent decades, and the age of first marriage has delayed [7]. The average age of a woman’s first marriage has increased from 26.3 years in 1999 to 28.7 years in 2009 and to 30.8 years in 2020 [8]. Along with this marriage trend, the average age of first childbirth was 32.2 years, and the total birth rate was 0.84 in 2020, the lowest among Organization for Economic Cooperation and Development (OECD) countries [8]. This phenomenon may be a reason why young adult women are not adhering to cervical cancer screening. More specifically, adherence to cervical cancer screening among women in their 20s and 30s was 20.9% and 48.5% in 2018, respectively, which is lower than that of other age groups in Korea [5]. Compared to the adherence to cervical cancer screening in developed countries, including the United States (82.9% in women aged 21–40 years) and England (72.5% in women aged 25–40 years), the adherence in Korea is much lower [9,10].

However, there is insufficient evidence on whether marriage affects adherence to cervical cancer screening among young adult women in Korea. Therefore, this study aimed to evaluate the association between marriage and childbirth with adherence to cervical cancer screening among women in their 20s and 30s.

## 2. Materials and Methods

### 2.1. Study Design and Participants

The current data were collected from the cross-sectional Korean National Cancer Screening Survey (KNCSS). The KNCSS is intended to assess both organized and opportunistic cancer screening adherence and has monitored adherence annually since 2004 [11]. The eligible participants for the survey were those who had never been diagnosed with cancer. The sex- and age-specific inclusion criteria were determined based on the NCSP guidelines for the five major cancers, namely gastric, liver, colorectal, breast, and cervical cancers. To form the study sample, multistage stratified random sampling was performed according to the geographical area, age, and sex. The participants were recruited through door-to-door visits. In 2004, computer-assisted telephone interviews were conducted for the survey. However, since 2005, the survey has been conducted by trained professionals through face-to-face interviews during home visits; at least three home visits are attempted for these interviews. The professionals asked the participants for information on the survey items and filled out the survey questionnaire accordingly. A detailed explanation of the survey protocol is given elsewhere [5,11]. In this study, data on women aged 20–39 years between 2017 and 2020 were analyzed, because women in their 20s have been included in the survey since 2014 and the survey items on marriage and childbirth have been included since 2017. The response rate ranged from 27.5% to 45.6% (2017–2020). This study was approved by the Institutional Review Board of the National Cancer Center in Korea (approval number: NCC2019-0233). Prior to the survey, the participants verbally consented to participate in the survey for public purposes; the requirement of a written informed consent was waived. 

### 2.2. Measures

To measure adherence to cervical cancer screening, we used the recommended cervical cancer screening rate, defined as the percentage of women in the population eligible for screening who have had a cervical cancer screening (conventional Pap smear or liquid-based cytology) within the past two years depending on the screening interval of the Protocol of the National Cancer Screening Program for Cervical Cancer in Korea. The marital status was classified into unmarried and married (widowed (*n* = 1), divorced (*n* = 10), and currently married (*n* = 1230)). The number of births was classified into three categories (0, 1, ≥2) according to the number of children born.

We controlled for participants’ sociodemographic characteristics including age, self-rated health status, supplemental medical insurance for cancer, education level, monthly household income, and the number of family members. Self-rated health status was classified into bad (response to bad or very bad), fair (response to fair), good (response to good or very good). Supplemental medical insurance for cancer was classified into ‘yes’ and ‘no’ depending on whether they were privately insured against cancer. Education level was divided into high school graduation or less and college or higher. Monthly household income was characterized as <4000$, 4000–4990$, and ≥5000$. The number of family members was measured as the total number of family members living together including the participant, and was categorized into 1 (participant living alone), 2–3, and ≥4.

### 2.3. Statistical Analysis

The collected data were analyzed using SAS software (ver. 9.4; SAS Institute, Cary, NC, USA). We performed the Chi-square test to assess the participants’ characteristics according to marital status (unmarried vs. married). Statistical significance was defined as a *p*-value of <0.05. A simple logistic regression analysis was used to investigate the relationship between each variable and adherence to cervical cancer screening. A multiple logistic regression analysis adjusting for the covariates of age, self-rated health status, supplemental medical insurance for cancer, education level, monthly household income, and family member count was conducted to identify the association between marriage-related variables (marital status and number of births) and cervical cancer screening.

## 3. Results

A total of study number of 3925 was comprised of 2350 unmarried women and 1575 married women (Table 1). Of the total, 39.1% adhered to cervical cancer guidelines. Among unmarried and married women, 26.6% and 57.7%, respectively, adhered to cervical cancer screening guidelines. Approximately 80% of unmarried women were in their 20s, and only two unmarried women had experienced childbirth. Most of the married women consisted of women in their 30s, with 44.5% and 38.2% of married women having given birth once and more than twice, respectively.

The results of the logistic regression analysis are presented in Table 2. The model fit, as calculated using the Hosmer-Lemeshow goodness-of-fit test, was good (Model I, χ^2^ = 11.02, *p*-value = 0.138; Model II, χ^2^ = 7.00, *p*-value = 0.536). We independently analyzed the association between marital status and cervical cancer screening (Model I), and marital status & number of births and cervical cancer screening (Model II). For multivariable logistic regression analysis, Models I and II were adjusted for covariates. In Model I, compared with unmarried women, married women were more likely to get cervical cancer screening (adjusted odds ratio [aOR] = 2.80, 95% confidence interval [CI]: 2.99–3.44). Among women in their 20s and 30s, the married women were more likely to get cervical cancer screening compared to unmarried women (aOR = 2.59, 95% CI: 1.75–3.85; aOR = 2.64, 95% CI: 2.00–3.49, respectively). In Model II, when compared to unmarried women, married women without childbirth experience, married women with one birth count, and married women with more than two births were more likely to undergo cervical cancer screening (aOR = 1.84, 95% CI: 1.38–2.46; aOR = 2.92, 95% CI: 2.27–3.75; aOR = 3.64, 95% CI: 2.80–4.75, respectively). As the number of births of married participants increased, the adherence to cervical cancer screening guidelines increased significantly (*p* for trend, <0.001); this trend was more pronounced in women aged 30–39 years.

## 4. Discussion

This study demonstrated that cervical cancer screening uptake was significantly higher among married women than among unmarried women. As the number of births for married women increased, the likelihood of getting cervical cancer screening also increased compared to unmarried women. These results suggest that marriage and childbirth are associated with young adult women visiting obstetrics and gynecology clinics and receiving screening for cervical cancer.

Considering marital status, married women were more likely to undergo cervical cancer screening than unmarried women; 57.7% of married women and 26.6% of unmarried women were screened for cervical cancer in this survey. Kaso et al. also examined the factors associated with the likelihood of getting cervical cancer screening in women aged 20–39 years. They reported that, compared to unmarried women, married women were 2.4 times more likely to have received screening in the two years prior to the study [6]. A national survey on Lithuanian women aged 25–44 years revealed that compared to unmarried women, the probability of not getting screened for cervical cancer within three years of eligibility prior to the survey was 0.44 times lower in married women [12]. Several studies have also reported this positive association between marriage and adherence to cervical cancer screening guidelines across all age groups [13,14,15,16,17]. For example, a study on a representative sample of American women aged 21–65 years reported that compared to unmarried women, married women or unmarried women living with their partners were 1.13 times more likely to have participated in cervical cancer screening during the three years prior to the study [17]. In a national survey conducted in France, the rates of adherence and non-adherence to cervical cancer screening among unmarried women aged 25–65 years who do not live with their partner in the three years prior to the survey were 30% and 40%, respectively; this difference was significant [16]. Furthermore, similar associations have been found in studies on colorectal, breast, and prostate cancer screening [13,18,19].

Also, compared to unmarried participants, cervical cancer screening rates in married women with children was significantly higher in our study. Moreover, among women in their 30s, adherence to the screening was somewhat higher in women with two or more children than in women with one child. Olesen et al. also reported that in the two years prior to their study, women with children participated in cervical cancer screening about twice as much as women without children [20]. A study conducted in Thailand reported that the reasons for not participating in cervical cancer screening in the five years preceding the survey were an absence of symptoms, single marital status, and not having children [14]. Marriage and childbirth in adult women could be the determinants for undertaking cervical cancer screening.

In addition, the age at first marriage has risen to the third decade of life, and birth rates are continuously decreasing among young adult women in Korea [7]. This trend was also observed in our study: 6.3% of the participants in their 20s and 75.3% of the participants in their 30s were married. Furthermore, 25.8% of participants in their 20s and 52.8% of those in their 30s were screened for cervical cancer (data not shown), showing a significant difference in our study. Before women in their 20s were included in the NCSP for cervical cancer, their rate of cervical cancer screening was much lower at 15% [5]. This low adherence in women in their 20s is similar to that of Asian countries, including China (13.7%) and Japan (25.0%) [21,22], but is quite different from the rates in developed Western countries [9,10]. In England, where the National Health Service’s cervical cancer screening is offered to women aged 25–64 years, the adherence to cervical cancer screening among the 25–29 age group was reported to be around 65% over the past decade [9]. A national health interview survey in the U.S. reported that screening rates among women who were aged 21–30 was 75.8% [10]. Therefore, based on our findings, efforts are urgently required to identify barriers to and reluctance to getting cervical cancer screening among unmarried young adult women in Korea. 

The reason for non-adherence to cervical cancer screening among unmarried women could be explained by the sociocultural context related to sexual behavior. Among unmarried Korean young women, sexual intercourse and visits to an obstetrics and gynecology clinic have often considered a stigma [23]. This sex-related closed culture is often seen in Asian countries as well. Gibson et al. reported that Asian American college women were less likely to talk about sex compared to White college women [24]. In Chinese culture, unawareness of the need for cervical cancer screening and sexual activity among young adults was one of the barriers to cervical cancer screening [25]. Moreover, in Japanese culture, for young adult women, the fear of seeing others when visiting obstetricians and gynecologists and the embarrassment of talking about sex, even if they talked to a doctor, was reported [26]. Meanwhile, this sociocultural context could be linked to inadequate sexual health education in adolescence. Most Korean schools have included sexual health education in their curriculums since 2009. However, Ahn et al. reported that only approximately 20% of those in their 20s received adequate sexual health education [27]. Also, a survey conducted in Japanese unmarried young adult women reported that approximately 30% of them knew about cervical cancer from school [26]. Hence, lack of knowledge toward cervical cancer screening is one of the greatest barriers to cervical cancer screening, especially in young adult women, [23]. The conservative sociocultural context of sexual behavior could further accelerate the decline in knowledge about cervical cancer and its screening and consequently lead to a decline in the uptake of screening.

While there have been interventional studies conducted in middle-aged women to increase rates of cervical cancer screening, to date, there have been few such studies conducted in young adult women [28]. Particularly, in women with low cervical cancer screening adherence, the cultural approaches significantly improved their knowledge level and screening adherence [29]. A recent meta-analysis reported that those who received a culturally tailored education (i.e., using lay health workers as instructors, culturally sensitive programs and materials, using cancer survivors) showed a 54% increase in cervical cancer screening adherence [30]. Also, vaginal self-sampling based on self-collection has recently been introduced as an alternative Pap smear, especially among non-adherent or under-attending women [31,32,33], which could be effective for unmarried young adult women who are reluctant to visit obstetrics and gynecology due to social stigma. Therefore, we suggest that culturally customized interventions addressing barriers of unmarried young adult women within the sociocultural context of Korea could help get them to undertake the screening.

The strength of our study is that, to the best of our knowledge, this study is the first to elucidate the influence of marriage on adherence to cervical cancer screening recommendations among young adult women through a representative sample in Korea. More noteworthy, as mentioned earlier, Korean young women are getting married later [7], and their first sexual experiences are also happening at a younger age [4], revealing that they may be more vulnerable to cervical cancer. Based on these findings, national policymakers will be able to select groups vulnerable to cervical cancer and come up with specific strategies to increase their screening rates.

### Limitations

Our study had several limitations. First, causal relationships could not be determined as the KNCSS data set had a cross-sectional design. However, our findings remain meaningful since the data were collected from nationally representative samples via stratified, multistage, random sampling according to geographical area, age, and sex. Second, our results do not rule out the possibility of respondent bias. Because we did not have information on participation in cervical cancer screening, marital status, and general characteristics for non-respondents, we could not compare baseline results between respondents and non-respondents. Third, our study confirmed that marriage and childbirth are factors that provide opportunities for cervical cancer screening, but a more important issue is whether subsequent cervical cancer screening is regularly performed after marriage and childbirth. Thus, further studies on issues related to regular screening after marriage and childbirth should be conducted.

## 5. Conclusions

Our study found that marriage and childbirth were significant factors associated with cervical cancer screening adherence among young adult women. Furthermore, adherence was significantly more likely to increase in married women with an increased number of births. The most important issue here is that most women in their 20s were unmarried with no childbirth experience, reflecting the recent trend of late marriage among young adult women in Korea. Therefore, unmarried women, particularly in their 20s, are the target population that be the should focus of increasing uptake rates of cervical cancer screening. More aggressive and tailored strategies should be carried out within the sociocultural context of Korea.

## Figures and Tables

**Table 1:** Participants’ characteristics.

Characteristics	Overall	Marital Status		*p*-Value
		Unmarried	Married	
	3925	2350 (59.9)	1575 (40.1)	
Cervical cancer screening				
No	2392 (60.9)	1725 (73.4)	667 (42.4)	<0.001
Yes	1533 (39.1)	625 (26.6)	908 (57.7)	
Age, years				
20–29	2000 (51.0)	1875 (79.8)	125 (7.9)	<0.001
30–39	1925 (49.0)	475 (20.2)	1450 (92.1)	
Number of births				
0	2621 (66.8)	2348 (99.9)	273 (17.3)	<0.001
1	703 (17.9)	2 (0.1)	701 (44.5)	
≥2	601 (15.3)	0 (0.0)	601 (38.2)	
Self-rated health status				
Bad	66 (1.7)	37 (1.6)	29 (1.8)	0.033
Fair	611 (15.6)	338 (14.4)	273 (17.3)	
Good	3248 (82.8)	1975 (84.0)	1273 (80.8)	
Supplemental medical insurance for cancer				
No	1014 (25.8)	782 (33.3)	232 (14.7)	<0.001
Yes	2911 (74.2)	1568 (66.7)	1343 (85.3)	
Education level				
≤High school	1081 (27.5)	658 (28.0)	423 (26.9)	0.444
≥College or higher	2844 (72.5)	1692 (72.0)	1152 (73.1)	
Monthly household income, USD ($) ^a^				
<4000	1326 (33.8)	665 (28.3)	661 (42.0)	<0.001
4000–4990	1095 (27.9)	598 (25.5)	497 (31.6)	
≥5000	1504 (38.3)	1087 (46.3)	417 (26.5)	
Number of family members ^b^				
1	334 (8.5)	318 (13.5)	16 (1.0)	<0.001
2–3	1720 (43.8)	808 (34.4)	912 (57.9)	
≥4	1871 (47.7)	1224 (52.1)	647 (41.1)	

^a^ 1 USD = 1000 KWN. ^b^ The number of family members refers to family members currently living with the participant, including the participant herself. That is, the number of family members of ‘1’ means that the participant lives alone. Data are n or n (%) unless otherwise specified.

**Table 2:** Association between marriage-related variables and cervical cancer screening.

Items	Cervical Cancer Screening (Yes vs. No)					
	Total (*n* = 3925)		20–29 Years (*n* = 2000)		30–39 Years (*n* = 1925)	
	cOR (95% CI)	aOR (95% CI)	cOR (95% CI)	aOR (95% CI)	cOR (95% CI)	aOR (95% CI)
MODEL I						
Marital status						
Unmarried	1.00 (Reference)	1.00 (Reference)	1.00 (Reference)	1.00 (Reference)	1.00 (Reference)	1.00 (Reference)
Married	3.76 (3.28–4.30)	2.80 (2.29–3.44)	3.08 (2.14–4.44)	2.59 (1.75–3.85)	2.49 (2.01–3.09)	2.64 (2.00–3.49)
MODEL II						
Marital status & number of births						
Unmarried ^a^	1.00 (Reference)	1.00 (Reference)	1.00 (Reference)	1.00 (Reference)	1.00 (Reference)	1.00 (Reference)
Married & 0	2.37 (1.83–3.05)	1.84 (1.38–2.46)	3.22 (2.01–5.15)	2.61 (1.59–4.28)	1.43 (1.02–2.00)	1.55 (1.06–2.26)
Married & 1	4.08 (3.42–4.86)	2.92 (2.27–3.75)	1.98 (0.95–4.10)	1.58 (0.74–3.39)	2.73 (2.14–3.49)	3.02 (2.19–4.15)
Married & ≥2	4.24 (3.51–5.11)	3.64 (2.80–4.75)	5.09 (2.10–12.35)	4.85 (1.95–12.07)	2.73 (2.12–3.50)	3.07 (2.15–4.37)
*p* for trend	<0.001	<0.001	<0.001	<0.001	<0.001	<0.001

For multivariable logistic analysis, Model I and Model II adjusted for age, self-rated health status, supplemental medical insurance for cancer, education, monthly household income, and family member. ^a^ Two of the unmarried women had a child. aOR, adjusted odds ratio; CI, confidence interval; cOR, crude odds ratio.

## Data Availability

The data presented in this study are available in this article.
